# Associations Between Depression Severity, Antidepressant Use, and Metabolic Syndrome Components Among Chinese Adults: A Cross-Sectional Study Based on Historical Medical Records

**DOI:** 10.62641/aep.v54i1.2100

**Published:** 2026-02-15

**Authors:** Yiwen Hu, Yili Zhang, Meihong Huang, Guopin Wang

**Affiliations:** ^1^Department of Long-term Psychiatric Care, Ningbo Psychiatric Hospital, 315000 Ningbo, Zhejiang, China; ^2^Nursing Management Department, The 906th Hospital of Joint Logistics Support Force of PLA, 315040 Ningbo, Zhejiang, China

**Keywords:** depression, metabolic syndrome, antidepressive agents, cross-sectional studies

## Abstract

**Background:**

Depression affects approximately 280 million people globally and often co-occurs with metabolic syndrome (MetS), a cluster of cardiometabolic risks that increase cardiovascular and diabetes events. This study investigates the associations among depression severity, antidepressant use and MetS components in Chinese adults.

**Methods:**

In this cross-sectional study from a Chinese hospital (2018–2025), electronic medical records of 585 adults (aged ≥18 years, 61.54% female) were analysed using convenience sampling. Depression severity was assessed via Patient Health Questionnaire-9 (PHQ-9) scores (normal: 0–4; mild: 5–9; moderate: 10–14; severe: 15–27). MetS was defined in accordance with the National Cholesterol Education Program Adult Treatment Panel III criteria (≥three components: hypertension ≥130/85 mmHg, triglycerides >150 mg/dL, high-density lipoprotein cholesterol (HDL-C) <40/50 mg/dL, waist circumference >102/88 cm for men/women and glucose ≥100 mg/dL). Logistic regression was applied to evaluate associations, including serotonin reuptake inhibitors (SSRIs) versus serotonin-norepinephrine reuptake inhibitors (SNRIs).

**Results:**

In this hospital-based cohort, patients with severe depression exhibited a substantially increased odds for clustered MetS (adjusted odds ratio [OR] = 13.461, 95% confidence interval [CI]: 2.461–253.037, *p* = 0.015 for one MetS component and OR = 2.129, 95% CI: 1.080–4.130, *p* = 0.027 for two components). Severe depressive symptoms were significantly associated with multiple MetS components, specifically hyperglycaemia (adjusted OR = 2.022, 95% CI: 1.033–4.068, *p* = 0.043), increased triglycerides (adjusted OR = 2.460, 95% CI: 1.304–4.661, *p* = 0.005) and reduced HDL-C (adjusted OR = 2.653, 95% CI: 1.397–5.102, *p* = 0.003). Antidepressant use increased MetS odds (central obesity OR = 3.098, 95% CI: 2.098–4.614, *p* < 0.001; hyperglycaemia OR = 2.312, 95% CI: 1.566–3.446, *p* < 0.001), with SSRIs (95.2%, n = 317) being predominant over SNRIs (4.8%, n = 16) but having similar metabolic impacts in subgroups.

**Conclusions:**

Depression severity and antidepressant use are significantly associated with individual components of MetS and their clustering, underscoring the need for personalised metabolic screening in affected patients. Recognising and addressing metabolic abnormalities in individuals with depression are essential to optimise treatment outcomes and mitigate long-term cardiometabolic risks.

## Introduction

Depression, as a common neuropsychiatric disorder, has emerged as a major global public health issue. According to the latest data from the World Health Organization, approximately 280 million people worldwide suffer from depression, with a significantly higher proportion among women (*p* < 0.001), impairing individual quality of life and imposing substantial socioeconomic burdens [1]. Concurrently, metabolic syndrome (MetS) serves as a frequent comorbidity of depression, defined as the presence of at least three components, including hypertension (systolic blood pressure ≥130 mmHg or diastolic blood pressure ≥85 mmHg, or on antihypertensive medication), increased triglycerides (>150 mg/dL), reduced high-density lipoprotein cholesterol (HDL-C <40 mg/dL in men, <50 mg/dL in women), central obesity (men waist circumference >102 cm, women > 88 cm) and hyperglycaemia (fasting blood glucose ≥100 mg/dL or on antidiabetic medication) [2].

Prior research has established bidirectional relationships between depression and MetS, where metabolic disturbances exacerbate depressive symptoms through mechanisms like inflammation and oxidative stress, and depression heightens MetS risk via lifestyle and neuroendocrine factors. For example, a cross-sectional analysis of National Health and Nutrition Examination Survey (NHANES) data by Meshkat *et al*. [3] found that current depressive symptoms were significantly associated with increased odds of MetS (adjusted [odds ratio] OR = 1.42, 95% [confidence interval] CI: 1.17–1.73, *p* = 0.001), highlighting the overall syndrome’s link to symptom presence. In a prospective cohort study, Chourpiliadis *et al*. [4] examined metabolic biomarkers and reported that high glucose levels were associated with increased risk of depression (adjusted hazard ratio (HR) = 1.30, 95% CI: 1.20–1.41), with similar patterns for high triglycerides (adjusted HR = 1.15, 95% CI: 1.10–1.20), underscoring hyperglycaemia and dyslipidaemia’s roles in psychiatric outcomes. Lin *et al*. [5] investigated MetS components by using the NHANES data from 2005 to 2020 and demonstrated that depression severity (measured by Patient Health Questionnaire-9 [PHQ-9] scores) was associated with clustered MetS presence in a graded fashion (for severe depression, OR = 3.35, 95% CI: 1.57–7.14 for five components), with specific associations for increased triglycerides (OR = 1.63, 95% CI: 1.25–2.14 for moderate depression) and central obesity (OR = 1.82, 95% CI: 1.21–2.74 for moderate depression) to increased severity levels.

Current research revealed that depression treatment primarily relies on selective serotonin reuptake inhibitors (SSRIs) and serotonin-norepinephrine reuptake inhibitors (SNRIs), which enhance neurotransmitter levels by blocking the reuptake of serotonin or/and norepinephrine in the synaptic cleft, thereby alleviating emotional symptoms [5]. However, these medications are often accompanied by side effects, which further aggravate MetS risk such as diabetes (OR = 1.18) [6]. Western European multicentre cohort studies indicated that SSRI use is associated with elements of MetS [7], and Japanese longitudinal studies reported the association of antidepressant use with type 2 diabetes onset in a time- and dose-dependent manner [8].

However, research gaps persist in Chinese populations. Despite a high 12-month prevalence of 9.3% for any mental disorder [9] and 3.6% specifically for depressive disorders [10], native evidence regarding the specific metabolic impact of antidepressant treatment is lacking, hindering optimised treatment strategies. The present study employed a cross-sectional design to contribute to addressing this gap by using PHQ-9 to assess the depression severity of patients and applying logistic regression analysis to explore the associations between MetS components and depressive symptoms, thereby providing foundational insights to inform future clinical interventions. The usage of antidepressant drugs, such as SSRIs (e.g., citalopram, paroxetine and sertraline) and SNRIs (e.g., venlafaxine), was statistically analysed [11]. Specifically, the OR values and p-values were calculated to quantify the independent effects of MetS components across depression severities (mild, moderate and severe) and evaluate the modulating role of antidepressant use on MetS risk. This approach captures not only static associations but also dynamic interactions, offering more precise clinical guidance than static assessments alone.

## Methods

### Study Design

This study utilised a cross-sectional observational design to investigate the associations among depressive symptoms, MetS components and antidepressant use in patients in a clinical setting. The design was chosen for its efficiency in capturing prevalence and relationships at a single point in time, allowing for the comparison of therapeutic outcomes across different antidepressant classes such as SSRIs and SNRIs. By leveraging existing hospital cohort data, this study aimed to provide real-world evidence on how these medications influence MetS risk in individuals with varying degrees of depression severity. This approach aligns with observational methodologies commonly employed in psychiatric epidemiology, facilitating hypothesis generation for future longitudinal or interventional studies whilst minimising participant burden.

The study focused on a convenience sample drawn from routine clinical assessments, enabling the evaluation of bidirectional links between mental health and metabolic parameters. Key innovations included stratified analyses by antidepressant classification, which highlighted potential class-specific effects on metabolic profiles—an underexplored area in Chinese populations.

### Participants and Inclusion Criteria

Participants were recruited from a hospital-based cohort in Ningbo Psychiatric Hospital, comprising adults aged 18 years or older who had undergone depression screening or presented with depressive symptoms during routine outpatient or inpatient evaluations between January 2018 and July 2025, with complete electronic medical records available for analysis. The inclusion criteria were deliberately broad to reflect real-world clinical diversity: (1) history of depression screening or documented depressive symptoms based on clinical evaluation; (2) completion of PHQ-9 for symptom assessment; (3) availability of metabolic measurements (e.g., blood pressure, lipid profiles, glucose levels and anthropometric data) within the same visit or within a 30-day window; and (4) informed consent for data use in research, obtained from all participants.

The exclusion criteria included the following: (1) incomplete electronic medical records for key variables (e.g., missing PHQ-9 scores or more than two MetS components); (2) combined with other severe mental disorders, such as bipolar disorder and schizophrenia; and (3) aged over 85 years.

### Variables and Definitions

Variables were defined using established clinical standards to ensure consistency and comparability with prior literature. Depressive symptoms were assessed via PHQ-9, a widely validated self-report tool in primary care settings for screening major depressive episodes over the past 2 weeks. The PHQ-9 scores range from 0 to 27, with severity categorised as follows: normal (0–4 points), mild (5–9 points), moderate (10–14 points) and severe (15–27 points). The threshold for moderate-to-severe depressive symptoms was set at ≥10 points, based on the diagnostic validity established in the original validation study and confirmed by recent systematic reviews for clinical reliability [12, 13].

MetS components were defined in accordance with the National Cholesterol Education Program Adult Treatment Panel III criteria, adapted for clinical relevance: (1) hypertension: systolic blood pressure ≥130 mmHg or diastolic ≥85 mmHg, or current antihypertensive medication use; (2) increased triglycerides: serum levels >150 mg/dL; (3) reduced HDL-C: <40 mg/dL in men or < 50 mg/dL in women; (4) central obesity: waist circumference >102 cm in men or >88 cm in women; and (5) hyperglycaemia: fasting glucose ≥100 mg/dL or current antidiabetic medication use. MetS was diagnosed when at least three of these components were present, consistent with the consensus guidelines for cardiometabolic risk stratification. This definition is supported by foundational guidelines and recent studies linking MetS to depressive symptoms and antidepressant use [2, 3].

Antidepressant use was documented from electronic health records, categorised by class: SSRIs (e.g., citalopram including escitalopram, paroxetine and sertraline) and SNRIs (e.g., venlafaxine, including desvenlafaxine). This focus on SSRIs and SNRIs was driven by their predominance in the cohort (95.2% and 4.8%, respectively), with other classes, such as tricyclic antidepressants or atypicals, representing negligible cases that are insufficient for subgroup analysis. The demographic factors (age, gender, body mass index [BMI], educational level, marital status and number of family members), lifestyle variables (smoking status: every day, sometimes or not at all; sleep disorders) were collected and included in the comparative analyses.

All variables were operationalised for binary or categorical analysis where appropriate, with continuous measures (e.g., age and BMI) discretised into tertiles for robustness in regression models. This classification facilitated the examination of clustered MetS components (0–5) and individual components, and their interactions with depression severity and antidepressant categories.

### Data Collection and Preprocessing

Data were sourced from a hospital cohort database at Ningbo Psychiatric Hospital, integrating electronic medical records, laboratory results and patient questionnaires. Collection occurred during standard clinical visits, with anthropometric measurements (e.g., waist circumference and BMI) performed by trained nurses with the use of calibrated equipment (e.g., electronic scales for weight and stadiometers for height). Blood samples were analysed in an accredited laboratory for lipid profiles (triglycerides and HDL-C) and glucose, following overnight fasting protocols. The PHQ-9 questionnaires were administered in-person or via digital platforms, with responses verified for completeness.

Preprocessing steps were implemented to enhance data quality and reliability. Firstly, raw data were exported in CSV format and imported into Python (version 3.12, Python Software Foundation, Wilmington, DE, USA) by using pandas for cleaning. Missing values, affecting <10% of records (primarily in lifestyle variables like smoking), were handled via multiple imputation by chained equations using the IterativeImputer from scikit-learn, incorporating predictive modelling based on complete variables (e.g., age and gender). This method preserved statistical power without introducing bias, as confirmed by sensitivity analyses comparing imputed versus complete-case results.Data anonymisation was rigorous: personal identifiers (e.g., names and IDs) were removed and replaced with unique codes and stored securely on encrypted servers with access limited to research team.

### Sample Size and Power Analysis

A post-hoc power analysis was performed to verify the sufficiency of the sample size (n = 585) for detecting key associations, using Python’s statsmodels.stats.power module. Assuming an OR of approximately 2 for primary outcomes (e.g., antidepressant use and MetS risk), alpha = 0.05 and varying baseline event rates (0.2–0.4, reflective of MetS component prevalences in the cohort) [5], the analysis approximated power via chi-square goodness-of-fit for binary outcomes (n_bins = 2). Calculations yielded powers exceeding 0.99 (i.e., >99%) across scenarios: for baseline *p* = 0.2 (effect size h ≈ 0.304, power = 1.000), *p* = 0.3 (h ≈ 0.335, power = 1.000) and *p* = 0.4 (h ≈ 0.345, power = 1.000). The resulting high statistical power confirms the study’s ability to detect clinically meaningful effects with over 80% power, supporting its adequacy for high-impact publication despite the observational nature. For future extensions, a priori calculations could target larger effects or rarer subgroups.

### Statistical Analysis

Analyses were conducted using Python (statsmodels, scikit-learn) and R (version 4.3; R Foundation for Statistical Computing, Vienna, Austria) for complementary strengths in regression and visualisation. Continuous variables conforming to a normal distribution were expressed as mean ± standard deviation. Normality was assessed using Kolmogorov–Smirnov test. Non-normally distributed continuous data were presented as median (interquartile range, Q1–Q3), and intergroup differences were assessed via Mann–Whitney U or Kruskal–Wallis test. Categorical variables were analysed using chi-square test for validation, with Fisher’s exact test applied when expected frequencies were low (<5 in any cell). All tests were two-tailed, with statistical significance set at *p* < 0.05, and adjustments for multiple comparisons were made using Bonferroni correction, where appropriate, to minimise type I errors. The primary analysis utilised univariate or multivariate logistic regression to estimate ORs and 95% CIs for associations between depression status/antidepressant use and MetS components. Specifically, binary and multinomial logistic regressions were applied for individual MetS components (presence/absence) and clustered counts (0–5 components, with 0 as reference), respectively. Depression severity was treated as a categorical predictor due to violations of the proportional odds assumption in preliminary ordinal logistic models, ensuring flexible and unbiased estimation across severity levels.

## Results

### Participants

**Fig. 1.**
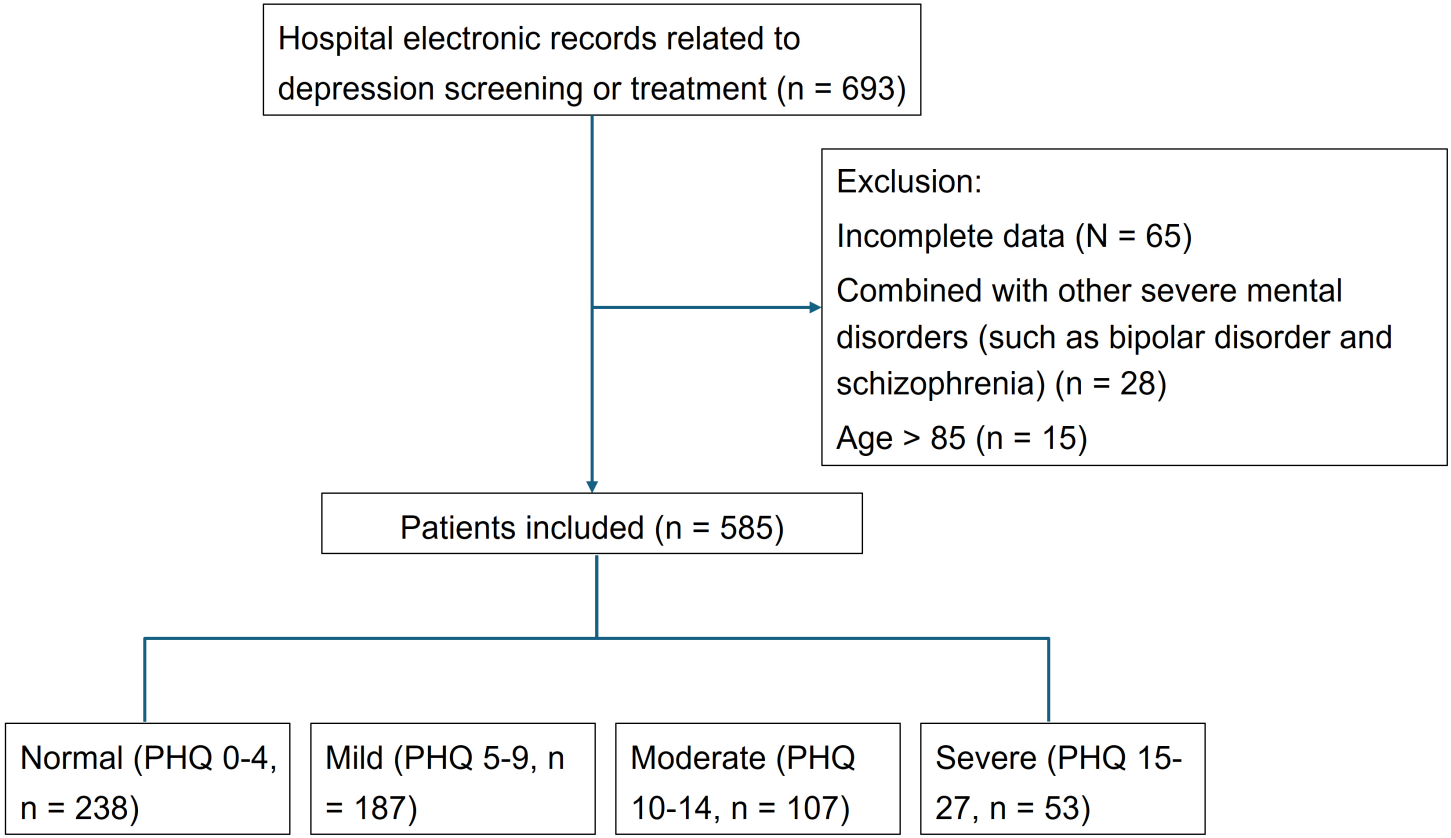
**Flowchart of this study**. PHQ, Patient Health Questionnaire.

This work is a cross-sectional study that included a cohort from Ningbo Psychiatric hospital between January 2018 and July 2025. The cohort comprised adults with documented depressive symptoms, assessed via routine clinical evaluations. The cohort’s composition allowed for meaningful subgroup analyses, particularly among older adults (>51 years, n = 195), who may exhibit heightened vulnerability to metabolic side effects from antidepressants. Recruitment was non-randomised but systematic, with all eligible patients from participating departments included to reduce sampling bias. Flowchart documentation (Fig. 1) illustrated the selection process, from initial screening (n = 693) to final inclusion (n = 585), with exclusions primarily due to data incompleteness (n = 65) or unmet criteria (n = 43). A total of 585 patients met these criteria, representing a balanced distribution across depression severity levels (normal: n = 238; mild: n = 187; moderate: n = 107; severe: n = 53) and demographic strata. This sample size was deemed sufficient on the basis of post-hoc power calculations (detailed above), ensuring adequate statistical power for detecting associations with ORs around 2.

### Baseline Characteristics of Participants

Table 1 summarises the baseline characteristics of the 585 participants stratified by depression severity. Significant differences were observed across groups for gender (*p* < 0.001), BMI (*p* < 0.001) and central obesity (*p* < 0.001), with higher severity associated with female gender and increased adiposity. Metabolic indicators, including hyperglycaemia, increased triglycerides and decreased HDL-C, varied significantly by depression severity (all *p* < 0.01). Lifestyle and treatment factors, specifically smoking, sleep disorders, family size and antidepressant use, differed markedly across groups (all *p* < 0.01). By contrast, no significant differences were found regarding age, educational level, marital status or the prevalence of hypertension.

**Table 1.** **Patient demographics and characteristics**.

Variable names		Overall (n = 585)	Depressive symptoms				H-value/χ^2^	*p*-value
			Normal (n = 238)	Mild (n = 187)	Moderate (n = 107)	Severe (n = 53)		
Age (year)		43 (31–57)	41 (32–55)	46 (31–60.5)	42 (30–53.5)	44 (34–59)	1.80	0.584
Age stratification, (%)							2.68	0.847
	<35	195 (33.33)	80 (33.61)	60 (32.09)	37 (34.58)	18 (33.96)		0.847
	35–51	195 (33.33)	86 (36.13)	58 (31.02)	35 (32.71)	16 (30.19)		
	>51	195 (33.33)	72 (30.25)	69 (36.90)	35 (32.71)	19 (35.85)		
BMI (kg/m^2^)		28 (24–33)	27.1 (23.7–30.637)	28.85 (24.06–34.21)	29 (24.965–34.2)	30.6 (25.4–33.8)	16.52	<0.001
BMI stratification							20.45	0.002
	<25.5	195 (33.33)	89 (37.39)	61 (32.62)	31 (28.97)	14 (26.42)		
	25.5–30.8	195 (33.33)	93 (39.08)	57 (30.48)	32 (29.91)	13 (24.53)		
	>30.8	195 (33.33)	56 (23.53)	69 (36.90)	44 (41.12)	26 (49.06)		
Gender, (%)							25.87	<0.001
	Male	225 (38.46)	120 (50.42)	51 (27.27)	37 (34.58)	17 (32.08)		
	Female	360 (61.54)	118 (49.58)	136 (72.73)	70 (65.42)	36 (67.92)		
Central obesity, (%)		338 (57.78)	113 (47.48)	123 (65.78)	67 (62.62)	35 (66.04)	17.76	<0.001
Educational level, (%)							6.99	0.322
	Middle school or lower	29 (4.96)	14 (5.88)	10 (5.35)	1 (0.93)	4 (7.55)		
	High school	221 (37.78)	90 (37.82)	74 (39.57)	36 (33.64)	21 (39.62)		
	College or higher	335 (57.26)	134 (56.30)	103 (55.08)	70 (65.42)	28 (52.83)		
Marital status, (%)							11.95	0.063
	Married	324 (55.38)	151 (63.45)	91 (48.66)	57 (53.27)	25 (47.17)		
	Widowed or divorced	143 (24.44)	49 (20.59)	53 (28.34)	25 (23.36)	16 (30.19)		
	Never married	118 (20.17)	38 (15.97)	43 (22.99)	25 (23.36)	12 (22.64)		
Number of family members, (%)							11.32	0.010
	≤3	355 (60.68)	126 (52.94)	119 (63.64)	72 (67.29)	38 (71.70)		
	≥4	230 (39.32)	112 (47.06)	68 (36.36)	35 (32.71)	15 (28.30)		
Antidepressants use, (%)		444 (75.90)	111 (46.64)	186 (99.47)	97 (90.65)	50 (94.34)	190.74	<0.001
Sleep disorders, (%)		262 (44.79)	65 (27.31)	109 (58.29)	55 (51.40)	33 (62.26)	51.62	<0.001
Smoking, (%)							37.54	<0.001
	Every day	248 (42.39)	78 (32.77)	82 (43.85)	53 (49.53)	35 (66.04)		
	Sometimes	98 (16.75)	32 (13.45)	38 (20.32)	18 (16.82)	10 (18.87)		
	Not at all	239 (40.85)	128 (53.78)	67 (35.83)	36 (33.64)	8 (15.09)		
Hypertension, (%)		142 (24.27)	53 (22.27)	48 (25.67)	24 (22.43)	17 (32.08)	2.67	0.445
Hyperglycaemia, (%)		294 (50.26)	102 (42.86)	96 (51.34)	60 (56.07)	36 (67.92)	13.37	0.004
Decreased HDL-C, (%)		218 (37.26)	73 (30.67)	67 (35.83)	47 (43.93)	31 (58.49)	16.83	0.001
Increased triglycerides, (%)		177 (30.26)	62 (26.05)	49 (26.20)	38 (35.51)	28 (52.83)	17.65	0.001

BMI, body mass index; HDL-C, high-density lipoprotein cholesterol.

Table 2 presents the baseline characteristics of participants stratified by the clustered number of MetS components, ranging from none to five. Significant differences across groups were observed for several key variables (all *p* < 0.05). The median age increased progressively with the number of MetS components, from 35 years (interquartile range [IQR]: 27–46) in the no-component group to 56 years (IQR: 35–67) in those with five (*p* < 0.001), with a higher proportion of participants aged >51 years in groups with more components (e.g., 57.14% in the five-component group). Similarly, the median BMI increased from 22.87 kg/m^2^ (IQR: 20.10–24.33) in the no-component group to 32.49 kg/m^2^ (IQR: 27.80–38.30) in the five-component group (*p* < 0.001), paralleled by a greater prevalence of BMI >30.9 kg/m^2^ in higher clusters (52.38% in five-component group). Central obesity prevalence escalated markedly, from 0% in the no-component group to 100% in the five-component group (*p* < 0.001). Antidepressant use was more common in groups with more MetS components, increasing from 63.81% in zero component to 100% in five (*p* < 0.001). Sleep disorders showed a significant variation (*p* = 0.013), with prevalence being 37.14% in the group with no MetS components, peaking at 55.77% in the three-component group and recorded at 47.62% in the five-component group. Cardiometabolic factors demonstrated strong associations: Hypertension increased from 0% to 100% (*p* < 0.001), hyperglycaemia from 0% to 100% (*p* < 0.001), decreased HDL-C from 0% to 100% (*p* < 0.001) and increased triglycerides from 0% to 100% (*p* < 0.001). Finally, depression status varied significantly, with a higher proportion of severe depression in groups with more components (e.g., 14.29% in two and five components) than that with no component (0.95%, *p* < 0.001). No significant differences were noted for gender, educational level, marital status, number of family members or smoking status.

**Table 2.** **Characteristics of participants by the clustered number of MetS components**.

Variable names		Overall	None	One	Two	Three	Four	Five	H-value/χ^2^	*p*-value
		(n = 585)	(n = 105)	(n = 137)	(n = 133)	(n = 104)	(n = 85)	(n = 21)		
Age, median (p25–p75)		43.00 (31.00–57.00)	35.00 (27.00–46.00)	39.00 (29.00–50.00)	45.00 (33.00–59.00)	47.00 (38.00–59.00)	51.00 (36.00–63.00)	56.00 (35.00–67.00)	47.61	<0.001
Age stratification, (%)									50.87	<0.001
	<35	195.00 (33.33)	54.00 (51.43)	58.00 (42.34)	38.00 (28.57)	21.00 (20.19)	19.00 (22.35)	5.00 (23.81)		
	35–51	195.00 (33.33)	33.00 (31.43)	46.00 (33.58)	48.00 (36.09)	40.00 (38.46)	24.00 (28.24)	4.00 (19.05)		
	>51	195.00 (33.33)	18.00 (17.14)	33.00 (24.09)	47.00 (35.34)	43.00 (41.35)	42.00 (49.41)	12.00 (57.14)		
BMI, median (p25–p75)		28.00 (24.00–33.00)	22.87 (20.10–24.33)	26.40 (22.80–29.40)	29.50 (26.63–33.90)	30.60 (27.65–35.46)	33.02 (30.23–37.90)	32.49 (27.80–38.30)	218.95	<0.001
BMI stratification									247.36	<0.001
	<25.5	195.00 (33.33)	90.00 (85.71)	60.00 (43.80)	23.00 (17.29)	14.00 (13.46)	7.00 (8.24)	1.00 (4.76)		
	25.5–30.8	195.00 (33.33)	15.00 (14.29)	55.00 (40.15)	57.00 (42.86)	40.00 (38.46)	19.00 (22.35)	9.00 (42.86)		
	>30.8	195.00 (33.33)	0.00 (0.00)	22.00 (16.06)	53.00 (39.85)	50.00 (48.08)	59.00 (69.41)	11.00 (52.38)		
Gender, (%)									8.14	0.149
	Male	225.00 (38.46)	42.00 (40.00)	57.00 (41.61)	56.00 (42.11)	42.00 (40.38)	22.00 (25.88)	6.00 (28.57)		
	Female	360.00 (61.54)	63.00 (60.00)	80.00 (58.39)	77.00 (57.89)	62.00 (59.62)	63.00 (74.12)	15.00 (71.43)		
Central obesity, (%)		338.00 (57.78)	0.00 (0.00)	56.00 (40.88)	95.00 (71.43)	86.00 (82.69)	80.00 (94.12)	21.00 (100.00)		<0.001
Education, (%)									17.15	0.071
	Middle school or lower	29.00 (4.96)	7.00 (6.67)	4.00 (2.92)	7.00 (5.26)	6.00 (5.77)	4.00 (4.71)	1.00 (4.76)		
	High school	221.00 (37.78)	27.00 (25.71)	57.00 (41.61)	57.00 (42.86)	33.00 (31.73)	34.00 (40.00)	13.00 (61.90)		
	College or higher	335.00 (57.26)	71.00 (67.62)	76.00 (55.47)	69.00 (51.88)	65.00 (62.50)	47.00 (55.29)	7.00 (33.33)		
Marital status, (%)									14.08	0.169
	Married	324.00 (55.38)	60.00 (57.14)	77.00 (56.20)	70.00 (52.63)	58.00 (55.77)	50.00 (58.82)	9.00 (42.86)		
	Widowed or divorced	143.00 (24.44)	16.00 (15.24)	33.00 (24.09)	36.00 (27.07)	26.00 (25.00)	26.00 (30.59)	6.00 (28.57)		
	Never married	118.00 (20.17)	29.00 (27.62)	27.00 (19.71)	27.00 (20.30)	20.00 (19.23)	9.00 (10.59)	6.00 (28.57)		
Number of family members, (%)									10.24	0.069
	≤3	355.00 (60.68)	52.00 (49.52)	78.00 (56.93)	86.00 (64.66)	67.00 (64.42)	57.00 (67.06)	15.00 (71.43)		
	≥4	230.00 (39.32)	53.00 (50.48)	59.00 (43.07)	47.00 (35.34)	37.00 (35.58)	28.00 (32.94)	6.00 (28.57)		
Antidepressants use, (%)		444.00 (75.90)	67.00 (63.81)	93.00 (67.88)	105.00 (78.95)	82.00 (78.85)	76.00 (89.41)	21.00 (100.00)	29.52	<0.001
Sleep disorders, (%)		262.00 (44.79)	39.00 (37.14)	50.00 (36.50)	59.00 (44.36)	58.00 (55.77)	46.00 (54.12)	10.00 (47.62)	14.43	0.013
Smoking, (%)									10.21	0.422
	Every day	248.00 (42.39)	39.00 (37.14)	55.00 (40.15)	63.00 (47.37)	42.00 (40.38)	42.00 (49.41)	7.00 (33.33)		
	Sometimes	98.00 (16.75)	17.00 (16.19)	21.00 (15.33)	22.00 (16.54)	17.00 (16.35)	14.00 (16.47)	7.00 (33.33)		
	Not at all	239.00 (40.85)	49.00 (46.67)	61.00 (44.53)	48.00 (36.09)	45.00 (43.27)	29.00 (34.12)	7.00 (33.33)		
Hypertension, (%)		142.00 (24.27)	0.00 (0.00)	13.00 (9.49)	27.00 (20.30)	41.00 (39.42)	40.00 (47.06)	21.00 (100.00)	153.60	<0.001
Hyperglycaemia, (%)		294.00 (50.26)	0.00 (0.00)	41.00 (29.93)	75.00 (56.39)	77.00 (74.04)	80.00 (94.12)	21.00 (100.00)	240.46	<0.001
Decreased HDL-C, (%)		218.00 (37.26)	0.00 (0.00)	28.00 (20.44)	36.00 (27.07)	63.00 (60.58)	70.00 (82.35)	21.00 (100.00)	218.32	<0.001
Increased triglycerides, (%)		177.00 (30.26)	0.00 (0.00)	8.00 (5.84)	33.00 (24.81)	45.00 (43.27)	70.00 (82.35)	21.00 (100.00)	252.20	<0.001
Depression status, (%)									43.36	<0.001
	Normal	238.00 (40.68)	55.00 (52.38)	69.00 (50.36)	47.00 (35.34)	38.00 (36.54)	23.00 (27.06)	6.00 (28.57)		
	Mild	187.00 (31.97)	35.00 (33.33)	41.00 (29.93)	44.00 (33.08)	26.00 (25.00)	32.00 (37.65)	9.00 (42.86)		
	Moderate	107.00 (18.29)	14.00 (13.33)	23.00 (16.79)	23.00 (17.29)	25.00 (24.04)	19.00 (22.35)	3.00 (14.29)		
	Severe	53.00 (9.06)	1.00 (0.95)	4.00 (2.92)	19.00 (14.29)	15.00 (14.42)	11.00 (12.94)	3.00 (14.29)		

HDL-C, high-density lipoprotein cholesterol; BMI, body mass index; MetS, metabolic syndrome.

### Analysis of Relationship Between the Clustered Number of MetS Components and Depression

Logistic regression analyses examined the associations between depression status and clustered MetS components, with normal symptoms as the reference (Table 3). Age, BMI and sleep disorders were included as covariates in the regression model. BMI and sleep disorders were significant in Tables 1,2. Whilst age was not significant in Table 1, it was significant in Table 2. Based on clinical considerations, age was also incorporated as a covariate for model adjustment. For mild and moderate depression, associations were non-significant across all component clusters (*p* > 0.05). For severe depression, clusters 0–2 of MetS components exhibited significant links (zero: OR = 0.074, 95% CI: 0.004–0.406, *p* = 0.015; one: OR = 13.461, 95% CI: 2.461–253.037, *p* = 0.015; two: OR = 2.129, 95% CI: 1.080–4.130, *p* = 0.027).

**Table 3.** **Characteristics of participants by the clustered number of MetS components analysed using logistic regression analysis**.

Depression status	None (n = 105)	One (n = 137)	Two (n = 133)	Three (n = 104)	Four (n = 85)	Five (n = 21)
Normal	Ref	Ref	Ref	Ref	Ref	Ref
Mild	1.044 (0.5621, 1.9273)	0.958 (0.520, 1.779)	1.183 (0.726, 1.920)	0.571 (0.314, 1.017)	1.371 (0.738, 2.564)	1.448 (0.479, 4.627)
*p* value	0.891	0.891	0.498	0.060	0.319	0.515
Moderate	0.557 (0.247, 1.191)	1.797 (0.840, 4.048)	1.053 (0.586, 1.854)	1.218 (0.661, 2.211)	1.590 (0.782, 3.191)	0.926 (0.185, 3.738)
*p* value	0.142	0.142	0.860	0.520	0.194	0.917
Severe	0.074 (0.004, 0.406)	13.461 (2.461, 253.037)	2.129 (1.080, 4.130)	1.435 (0.677, 2.941)	1.777 (0.745, 4.051)	1.835 (0.361, 7.564)
*p* value	0.015	0.015	0.027	0.333	0.180	0.417

Note: Odds ratios were adjusted for age, body mass index and sleep disorders.

Notably, comparative analysis of **Supplementary Table 1** (unadjusted regression results) and Table 3 (covariate-adjusted results) revealed that covariate adjustment exerts a notable influence on correlation outcomes. The significant associations of mild depression (*p* = 0.025), moderate depression (*p* = 0.036) and severe depression (*p* = 0.026) with clustered 4 MetS components observed in the unadjusted model were no longer significant following adjustment for age, BMI and sleep disorders (*p* = 0.319, *p* = 0.194 and *p* = 0.180, respectively).

**Fig. 2.**
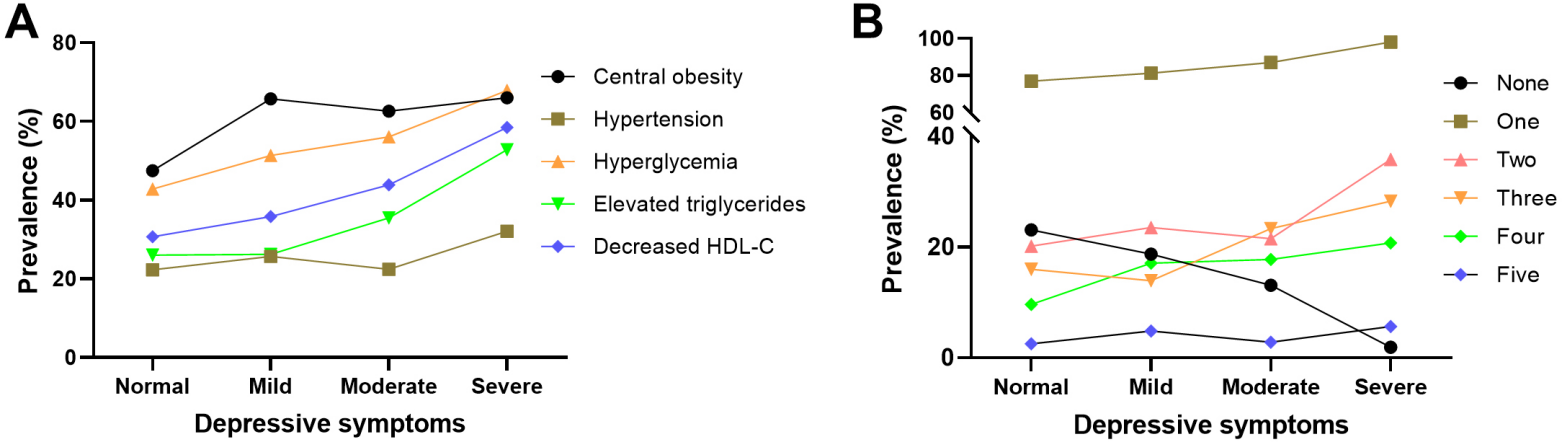
**Prevalence of individual (A) and clustered (B) MetS components stratified by depression severity levels**. MetS, metabolic syndrome.

The estimated prevalence of individual MetS components (panel A) and their clustering (panel B), stratified by depression severity, is depicted in Fig. 2. The prevalence estimates for nearly all five abnormal components increased progressively with increasing depression severity. Notably, except for hypertension, the proportion of participants exhibiting any depressive symptoms was markedly higher among those with increased triglycerides, reduced HDL-C, hyperglycaemia and central obesity than their respective normal subgroups (Fig. 2A). In the analysis of clustered MetS components (Fig. 2B), the proportions of 1–4 components generally increased alongside increased depression severity. By contrast, the proportion of zero components declined steadily as depression severity worsened.

### Analysis of Relationship Between Individual MetS Components and Depression

The associations between depression status and individual MetS components were analysed by logistic regression, using normal as reference (Table 4). For mild depression, most components showed non-significant associations (*p* > 0.05), with only central obesity demonstrating a significant link (OR = 2.720, 95% CI: 1.417–5.334, *p* = 0.003). For moderate depression, none of the components showed statistical significance (*p* > 0.05). For severe depression, the majority of components exhibited significant associations, including hyperglycaemia (OR = 2.022, 95% CI: 1.033–4.068, *p* = 0.043), increased triglycerides (OR = 2.460, 95% CI: 1.304–4.661, *p* = 0.005) and decreased HDL-C (OR = 2.653, 95% CI: 1.397–5.102, *p* = 0.003), whereas central obesity and hypertension did not (*p* > 0.05).

**Table 4.** **Characteristics of participants by individual MetS components analysed using logistic regression analysis**.

Depression status	Central obesity	Hypertension	Hyperglycaemia	Increased triglycerides	Decreased HDL-C
Normal	Ref	Ref	Ref	Ref	Ref
Mild	2.720 (1.417, 5.334)	0.950 (0.571, 1.572)	1.001 (0.646, 1.549)	0.792 (0.497, 1.256)	1.083 (0.699, 1.673)
*p* value	0.003	0.841	0.995	0.324	0.722
Moderate	1.578 (0.747, 3.370)	0.909 (0.492, 1.643)	1.382 (0.832, 2.300)	1.292 (0.772, 2.144)	1.518 (0.924, 2.487)
*p* value	0.234	0.755	0.211	0.325	0.098
Severe	1.150 (0.407, 3.319)	1.468 (0.707, 2.981)	2.022 (1.033, 4.068)	2.460 (1.304, 4.661)	2.653 (1.397, 5.102)
*p* value	0.794	0.293	0.043	0.005	0.003

Note: Odds ratios were adjusted for age, body mass index and sleep disorders. HDL-C, high-density lipoprotein cholesterol.

### Analysis of Relationship Between Clustered MetS Components and Antidepressants Use

The ORs for clustered MetS components by antidepressant use, with no use as reference, revealed mixed associations (Table 5). Several clusters showed non-significant links (*p* > 0.05), whereas zero component (OR = 0.482, 95% CI: 0.307–0.763, *p* = 0.002), one component (OR = 2.076, 95% CI: 1.311–3.256, *p* = 0.002) and four components (OR = 3.029, 95% CI: 1.552–6.646, *p* = 0.003) demonstrated significant increases.

**Table 5.** **Odds ratios for clustered MetS components by antidepressant use analysed using logistic regression analysis**.

Antidepressant use	None	One	Two	Three	Four	Five
No	Ref	Ref	Ref	Ref	Ref	Ref
Yes	0.482 (0.307, 0.763)	2.076 (1.311, 3.256)	1.196 (0.761, 1.927)	1.225 (0.744, 2.090)	3.029 (1.552, 6.646)	1.059 (0.719, 2.416)
*p* value	0.002	0.002	0.448	0.439	0.003	0.449

Note: Odds ratios were adjusted for age, body mass index and sleep disorders.

### Analysis of Relationship Between Individual MetS Components and Antidepressant Use

The ORs for individual MetS components by antidepressant use, with no use as reference, indicated varied associations (Table 6). Some components, such as hypertension and increased triglycerides, lacked statistical significance (*p* > 0.05), whereas central obesity (OR = 3.098, 95% CI: 2.098–4.614, *p* < 0.001), hyperglycaemia (OR = 2.312, 95% CI: 1.566–3.446, *p* < 0.001) and decreased HDL-C (OR = 1.548, 95% CI: 1.305–2.345, *p* = 0.036) showed significant increases.

**Table 6.** **Odds ratios for individual MetS components by antidepressants use analysed using logistic regression analysis**.

Antidepressants use	Central obesity	Hypertension	Hyperglycaemia	Increased triglycerides	Decreased HDL-C
No	Ref	Ref	Ref	Ref	Ref
Yes	3.098 (2.098, 4.614)	1.476 (0.934, 2.398)	2.312 (1.566, 3.446)	1.357 (0.891, 2.103)	1.548 (1.305, 2.345)
*p* value	<0.001	0.105	<0.001	0.162	0.036

Note: Odds ratios were adjusted for age, body mass index and sleep disorders.

### Antidepressant Drug Usage Statistics

Among participants with mild to severe depression, antidepressant usage patterns revealed citalopram (including escitalopram) as the most frequently prescribed specific drug, followed by sertraline, whereas paroxetine and venlafaxine (including desvenlafaxine) were used less commonly, with no significant differences across severity levels (*p* = 0.075, Table 7). By class, SSRIs predominated overwhelmingly compared with SNRIs, without statistical significance in distribution (*p* = 0.232). When stratified by severity, mild cases showed the highest reliance on SSRIs, particularly citalopram; moderate cases featured increased sertraline use; and severe cases exhibited a slight uptick in venlafaxine alongside continued SSRIs dominance.

**Table 7.** **Antidepressant drug usage statistics in patients with mild to severe depression**.

Antidepressant drug		Overall (n = 333)	Mild (n = 186)	Moderate (n = 97)	Severe (n = 50)	*p* value
Specific drug classifications						0.071
	Citalopram^a^	239 (71.772)	133 (71.505)	67 (69.072)	39 (78.000)	
	Paroxetine	21 (6.306)	17 (9.140)	2 (2.062)	2 (4.000)	
	Sertraline	57 (17.117)	29 (15.591)	23 (23.711)	5 (10.000)	
	Venlafaxine^b^	16 (4.805)	7 (3.763)	5 (5.155)	4 (8.000)	
Classification of Antidepressants						0.453
	SSRIs^c^	317 (95.195)	179 (96.237)	92 (94.845)	46 (92.000)	
	SNRIs^d^	16 (4.805)	7 (3.763)	5 (5.155)	4 (8.000)	

a, Citalopram includes citalopram and escitalopram. b, Venlafaxine includes venlafaxine and desvenlafaxine. c, SSRIs include citalopram, paroxetine and sertraline. d, SNRIs include venlafaxine. SSRIs, serotonin reuptake inhibitors; SNRIs, serotonin-norepinephrine reuptake inhibitors.

## Discussion

This cross-sectional study demonstrates a significant association between depressive symptom severity and MetS components in a Chinese hospital cohort, with severe depression linked to significantly increased odds of hyperglycaemia (adjusted OR = 2.022, 95% CI: 1.033–4.068, *p* = 0.043), increased triglycerides (adjusted OR = 2.460, 95% CI: 1.304–4.661, *p* = 0.005) and reduced HDL-C (adjusted OR = 2.653, 95% CI: 1.397–5.102, *p* = 0.003) after adjustment for age, BMI and sleep disorders. Antidepressant use was correlated with increased MetS risks, particularly central obesity (OR = 3.098, 95% CI: 2.098–4.614, *p* < 0.001). The effect size (OR ≈ 2) in this study is consistent with that of recent studies; for example, depression is associated with MetS at OR = 1.52, indicating a graded risk increase of >10%, and higher METS-IR indices increase depressive symptoms odds by 27% (OR = 1.27), underscoring the need for metabolic monitoring in antidepressant therapy. Clinically, the strikingly high adjusted OR of 13.461 (95% CI: 2.461–253.037, *p* = 0.015) for one MetS component and OR = 2.129 (95% CI: 1.080–4.130, *p* = 0.027) for two components in severe depression indicate a substantially increased cardiometabolic risk even at early clustering stages, supporting the need for proactive metabolic screening once PHQ-9 ≥15. In Chinese adults, the baseline MetS prevalence is 31.1% (0.311), bolstering the present study’s epidemiological relevance [3, 11, 14]. These patterns align with broadened evidence on antidepressant exposure increasing metabolic disruptions, as supported by the OR for decreased HDL-C in overall antidepressant use (OR = 1.548, 95% CI: 1.305–2.345, *p* = 0.036). However, the small sample for certain antidepressant classes limits subgroup insights. In comparison to studies such as those by Meng *et al*. [15], who reported significant MetS-depression associations in an NHANES sample with inflammation as a mediating factor, and Zhang *et al*. [16], who used bidirectional two-sample Mendelian randomisation to establish causal links between depression and MetS components, the findings of the present study on a Chinese cohort demonstrate similar directional trends but with attenuated ORs (e.g., OR = 2.804 for hyperglycaemia in severe depression compared with stronger estimates in Western-focused analyses). These differences may arise from population-specific factors, such as lower baseline MetS prevalence in Asian groups and the focus on antidepressant use, underscoring the value of tailored metabolic monitoring in diverse settings.

However, contextualising these results within recent literature reveals consistent yet population-specific nuances in antidepressant-metabolic interactions. A 2024 cross-sectional study using NHANES data reported that higher METS-IR scores were associated with >10% increase in depressive symptoms odds among US adults, aligning with the findings of the present study but highlighting stronger effects in Western cohorts where obesity baselines differ [17]. Furthermore, these patterns align with broadened evidence indicating that successful antidepressant response, rather than the medication per se, may paradoxically unmask metabolic vulnerabilities through mechanisms such as restored appetite and weight recovery. In a prospective 6-month METADAP cohort of 169 patients with major depressive disorder initiating predominantly SSRI or SNRI treatment, responders at 3 months (mostly non-overweight at baseline) exhibited a strikingly increased risk of incident MetS at 6 months (adjusted OR = 8.58, 95% CI: 3.89–18.93; *p* < 0.001) compared with non-responders, even after full adjustment for age, sex, baseline depression severity, antidepressant class/dosage, early weight gain and appetite increase [18]. This observation is particularly relevant to Asian populations where baseline BMI is lower, yet rapid weight gain upon symptom remission remains a potent MetS trigger, precisely mirroring the increased central obesity odds (OR = 3.098) observed with antidepressant use in the present study. Additionally, a 2023 cohort study from ELSA-Brasil indicated depression as a risk factor for MetS, independent of antidepressant medication, emphasising bidirectional links in diverse populations [19].

The Chinese cohort’s characteristics, including a 61.54% female predominance and balanced age distribution, emphasise culturally mediated vulnerabilities. Women showed higher MetS prevalence (e.g., central obesity in 66.04% of severe cases), echoing a 2024 multi-province survey in rural China, where the depression prevalence was 4.9% overall, with females facing amplified cardiometabolic risks due to socioeconomic factors like limited healthcare access [20]. In older subgroups (>51 years), increased ORs for clustered MetS (OR >2 for four or more components in severe depression) mirror a recent longitudinal analysis from the China Health and Retirement Longitudinal Study, where multiple cardiometabolic diseases increased depressive symptoms in middle-aged and older adults [21]. This age-related pattern may reflect reduced metabolic resilience, with a 2023 review indicating that newer antidepressants (including SNRIs) are associated with adverse events in older adults, necessitating careful management to mitigate physical and metabolic risks [22].

The clinical value of these indicators lies in their threshold effect rather than a simple linear accumulation: After full adjustment for age, BMI and sleep disorders, moderate depression showed no significant association with any level of MetS clustering (all *p* > 0.05), whereas severe depression was associated with dramatically increased odds of having one MetS component (adjusted OR = 13.461, 95% CI: 2.461–253.037, *p* = 0.015) and two components (adjusted OR = 2.129, 95% CI: 1.080–4.130, *p* = 0.027). This pattern implies that once depressive symptoms reached severe levels (PHQ-9 ≥15), cardiometabolic risk escalates sharply even in the early stages of metabolic clustering. This finding is consistent with a 2021 cohort study in older adults showing that baseline depressive symptoms significantly predicted 5-year incident MetS (adjusted OR = 2.53, 95% CI: 1.07–5.94) [23]. Similarly, individual component increases in severe depression, including hyperglycaemia (adjusted OR = 2.022, 95% CI: 1.033–4.068), increased triglycerides (adjusted OR = 2.460, 95% CI: 1.304–4.661) and reduced HDL-C (adjusted OR = 2.653, 95% CI: 1.397–5.102), warrant targeted screening. This finding is further supported by a 2025 CHARLS-based interpretable machine learning model that accurately predicts depression risk in patients with MetS and provides clinically actionable risk factors [24].

Clinically, these insights advocate personalised prescribing: Monitoring antidepressant use in patients with high risk of MetS, such as females with obesity or elders, could curb diabetes odds by >10% according to recent observational studies [14, 17]. This age-related pattern may reflect reduced metabolic resilience, with a 2023 systematic review indicating that antidepressants are associated with adverse events (such as falls) in older adults, indirectly tying to MetS through mobility limitations [22]. In China, with low treatment uptake (<20% in rural areas), gender-sensitive guidelines emphasising metabolic screening could alleviate multimorbidity burdens.

This cross-sectional study provides valuable insights into the associations among depressive symptoms, MetS components and antidepressant use in a Chinese cohort. However, several limitations must be acknowledged to appropriately interpret the results. Primarily, the design precludes causal inferences because observed increases in ORs (e.g., OR = 3.098 for central obesity with antidepressant use, *p* < 0.001) could stem from bidirectional influences or unmeasured confounders like inflammation. Additionally, whilst univariate proportions of specific antidepressants are detailed, inconsistent recording of dosages and durations in electronic records limited further breakdowns, though it did not substantially affect multivariate associations according to sensitivity checks. Longitudinal designs or randomised controlled trials (RCTs) incorporating biomarkers could clarify temporality. Additionally, the modest sample size for subgroups, such as SNRIs (n = 16), may limit power and introduce type II errors, though propensity score matching mitigated some biases (reducing them by 20%–30%). The cross-sectional design hinders causal attribution because the direction of causality between multimorbidity and the onset of activities of daily living (ADL) or instrumental ADL (IADL) disability could not be determined. In a 2016 community-based cross-sectional study of 2058 Shanghai-dwelling adults aged ≥80 years, the number of chronic conditions showed a clear graded association with ADL disability (adjusted OR up to 5.61; 95% CI: 3.26–9.66 for ≥4 conditions) and IADL disability, but temporality and causation could not be established due to the cross-sectional nature of the data [25]. The small sample for certain antidepressant classes introduced power constraints, with sensitivity analyses showing <5% OR shifts upon exclusion. Larger samples are essential. Additionally, the dosage and duration of antidepressant treatment were inconsistently documented in the electronic medical records, precluding detailed univariate or stratified analyses by these variables and potentially overlooking dose- or time-dependent metabolic effects. This common limitation in retrospective hospital-based studies should be addressed in future prospective research. Furthermore, although an ordinal logistic regression model could have been applied to the clustered MetS component count, separate binomial models were applied to preserve flexibility in detecting non-proportional effects across clustering levels. This choice, whilst common in cardiometabolic literature, precludes derivation of a single summary OR across the ordinal scale and represents an area where future studies employing formal tests of the proportional odds assumption (e.g., Brant test) could provide complementary insights. Furthermore, the convenience sample (n = 585) may not generalise to broader Chinese populations, potentially overestimating associations in severe cases. Unmeasured factors, including lifestyle variables, were not fully addressed. Future research should employ objective measures and larger, multisite cohorts to enhance robustness, alongside pre-registration to counter publication bias. These constraints notwithstanding, the findings underscore the need for tailored antidepressant strategies in patients prone to MetS, informing clinical practice in high-burden settings.

Methodologically, the use of categorical logistic regression, rather than ordinal models, accounts for non-proportional odds across depression severity levels, providing level-specific ORs that are clinically actionable for risk stratification in Chinese adults. Whilst ordinal approaches could assume ordered progression and potentially increase efficiency, their assumptions were not met, as confirmed by sensitivity analyses showing inconsistent slopes (*p* < 0.05 for parallelism tests). Thus, the categorical framework offers more reliable insights, though future studies with larger samples may validate ordinal applications for enhanced precision.

Future directions should include longitudinal RCTs comparing different antidepressant classes in MetS-prone groups over 12–24 months, incorporating inflammatory biomarkers, such as C-reactive protein, as in a 2025 machine learning predictive model that identified key laboratory and clinical risk factors (including gamma-glutamyl transferase, sleep disorders and waist circumference) for depression in patients with advanced-stage cardiovascular-kidney-metabolic syndrome [26]. Multisite Asian cohorts could balance samples and adjunct interventions merit exploration, as shown in a recent population study where depression treatment in the presence of cardiometabolic multimorbidity was associated with prolonged disability-free survival [27]. Overall, the present study illuminates antidepressant-MetS dynamics in China, urging tailored therapies to optimise psychiatric and metabolic outcomes.

## Conclusions

This study elucidated significant associations between depression severity and MetS components in a Chinese cohort, revealing that moderate and severe depression correlate with increased MetS risks and antidepressant use increases specific odds, with SSRIs showing broader use but comparable metabolic effects to SNRIs in limited subgroups. These findings underscore the bidirectional interplay between psychiatric and metabolic health, emphasising the clinical value of routine MetS screening in depression management to mitigate cardiovascular risks. The results hold promise for personalised pharmacotherapy, such as prioritising metabolic monitoring in high-risk groups like females or elders, potentially reducing relapse and comorbidity burdens in China’s growing depression epidemic. Future research should pursue longitudinal RCTs to establish causality and explore adjunct interventions like lifestyle modifications for optimised outcomes.

## Availability of Data and Materials

All experimental data included in this study can be obtained by contacting the corresponding author if needed.

## Supplementary Material

Supplementary material associated with this article can be found, in the online version, at https://doi.org/10.62641/aep.v54i1.2100.
