# Supplementary material for: Associations Between Depression Severity, Antidepressant Use, and Metabolic Syndrome Components Among Chinese Adults: A Cross-Sectional Study Based on Historical Medical Records
Source: Actas Esp Psiquiatr. 2026 Feb 15;54(1):107–20. doi: 10.62641/aep.v54i1.2100 (PMC12946717; doi:10.62641/aep.v54i1.2100)
Supplement: Supplementary file 1 [file ActEsp-54-1-107-120-s1.zip › Supplementary Table 1.docx]

Supplementary Table 1. Characteristics of participants by the clustered number of MetS components analyzed using unadjusted logistic regression analysis.

| Depression status | None (n= 105) | One (n = 137) | Two (n = 133) | Three (n = 104) | Four (n = 85) | Five (n = 21) |
| --- | --- | --- | --- | --- | --- | --- |
| Normal | Ref | Ref | Ref | Ref | Ref | Ref |
| Mild | 0.766 (0.473-1.227) | 1.305 (0.815, 2.113) | 0.218 (0.765, 1.936) | 0.850 (0.491, 1.452) | 1.930 (1.092, 3.460) | 1.955 (0.692, 5.927) |
| *p* value | 0.272 | 0.272 | 0.404 | 0.555 | 0.025 | 0.213 |
| Moderate | 0.501 (0.256-0.925) | 1.997 (1.081, 3.094) | 1.084 (0.612, 1.880) | 1.605 (0.903, 2.814) | 2.018 (1.038, 3.887) | 1.115 (0.232, 4.313) |
| *p* value | 0.034 | 0.034 | 0.778 | 0.102 | 0.036 | 0.879 |
| Severe | 0.064 (0.004, 0.302) | 15.628 (3.306, 279.626) | 2.212 (1.147, 4.189) | 2.078 (1.020, 4.096) | 2.448 (1.077, 5.310) | 2.320 (0.477, 9.109) |
| *p* value | 0.007 | 0.007 | 0.016 | 0.038 | 0.026 | 0.245 |
